# Interferon-γ Upregulates Expression of IFP35 Gene in HeLa Cells via Interferon Regulatory Factor-1

**DOI:** 10.1371/journal.pone.0050932

**Published:** 2012-12-04

**Authors:** Wei Yang, Juan Tan, Ruikang Liu, Xiaoxu Cui, Qinglin Ma, Yunqi Geng, Wentao Qiao

**Affiliations:** Key Laboratory of Molecular Microbiology and Biotechnology (Ministry of Education) and Key Laboratory of Microbial Functional Genomics (Tianjin), College of Life Sciences, Nankai University, Tianjin, China; University of Nebraska - Lincoln, United States of America

## Abstract

**Background:**

Interferon-induced 35-kDa protein (IFP35) plays important roles in antiviral defense and the progression of some skin cancer diseases. It can be induced by interferon-γ (IFN-γ) in multiple human cells. However, the mechanisms by which IFN-γ contributes to IFP35 induction remain to be elucidated.

**Methods/Principal Findings:**

We identified the transcription start sites of IFP35 by 5′ rapid amplification of cDNA ends (RACE) and cloned the promoter of IFP35. Sequence analysis and luciferase assays revealed two GC boxes and an IFN-stimulated response element (ISRE) in the 5′ upstream region of the transcription start sites, which were important for the basal transcription of IFP35 gene. Furthermore, we found that interferon regulatory factor 1 (IRF-1) and IRF-2 could bind to IFP35 promoter and upregulate endogenous IFP35 protein level. Depletion of endogenous IRF-1 by interfering RNA reduced the constitutive and IFN-γ-dependent expression of IFP35, whereas depletion of IRF-2 had little effect on IFN-γ-inducible IFP35 expression. Moreover, IRF-1 was recruited to the ISRE site in IFP35 promoter in IFN-γ treated HeLa cells, as demonstrated by electrophoretic mobility shift and chromatin immunoprecipitation assays.

**Conclusions/Significance:**

These findings provide the first evidence that IRF-1 and IRF-2 are involved in constitutive IFP35 expression in HeLa cells, while IRF-1 also activates IFP35 expression in an IFN-γ-inducible manner. Our data therefore identified a new IRF-1 and IRF-2 target gene, which may expand our current understanding of the versatile functions of IRF-1 and IRF-2.

## Introduction

Interferon (IFN)-induced 35-kDa protein (IFP35), an IFN-induced protein, was first isolated through differential screening of a cDNA library in IFN-γ treated HeLa cells. IFN-γ can induce IFP35 expression in various cells, including fibroblasts, monocytes/macrophages, and epithelial cells [1]. In addition, expression of IFP35 is also differentially regulated in the T cells of Sezary Syndrome patients and keratinocyes/skin of patients with atopic dermatitis (AD) [2], [3]. IFP35 contains a unique N-terminal leucine zipper motif and two C-terminal tandem Nmi/IFP35 homology domains (NIDs), which mediate the association between Nmi and IFP35 [4]. It can also interact with CKIP-1 (casein kinase 2-interacting protein-1) [5] and B-ATF (basic leucine zipper transcription factor, ATF-like) [6]. Additionally, we found previously that IFP35 confers resistance to bovine foamy virus (BFV) replication through the interaction with bovine Tas (BTas), a regulatory protein of BFV [7]. These protein-protein interactions suggest potential roles for IFP35 in host antiviral defense, cell apoptosis and other cytokine signaling pathways.

IFN-γ is a cytokine that plays important roles in a variety of biological processes including antiviral responses, anti-tumorigenesis, proinflammatory reactions and atherogenesis [8]. Signals of IFN-γ are transduced via two kinds of consensus sequences for IFN-γ response. One is the gamma-activated sequence (GAS), a binding site for the STAT1 homodimer [9], [10], [11], [12]. The other is IFN-stimulated regulatory element (ISRE), a binding site for IFN regulatory factors (IRFs) or IFN-stimulated gene factor 3 (ISGF3) [13].

The IFN regulatory factors (IRFs) are transcriptional mediators of IFN-induced signaling pathways [14]. To date, nine mammalian IRFs (IRF-1 to 9) have been identified and commonly possess a unique helix-turn-helix DNA-binding motif in the N-terminal region [15]. These factors can function as transcriptional activators or repressors. IRF-1 is the first identified member in the family and is induced upon IFN activation in many cell types [16], [17]. Upon IFN-γ stimulation, IRF-1 is transcriptionally regulated by STAT1 homodimer, which directs transcription of IRF-1 via the GAS element in the promoter [18]. As a transcriptional activator, IRF-1 directly binds to the ISRE that was found in the promoters of some IFN-regulated genes, including ISG20 [19], RANTES/CC15 [20] and LMP7 [21], and regulates their expression. IRF-2 was originally identified as a factor binding to the same recognition site as IRF-1 and was assumed to suppress the function of IRF-1 [17]. However, IRF2 also possesses a latent activation domain, and it was shown to activate several genes such as *histone H4* [22] and *gp91* [23].

**Figure 1:**
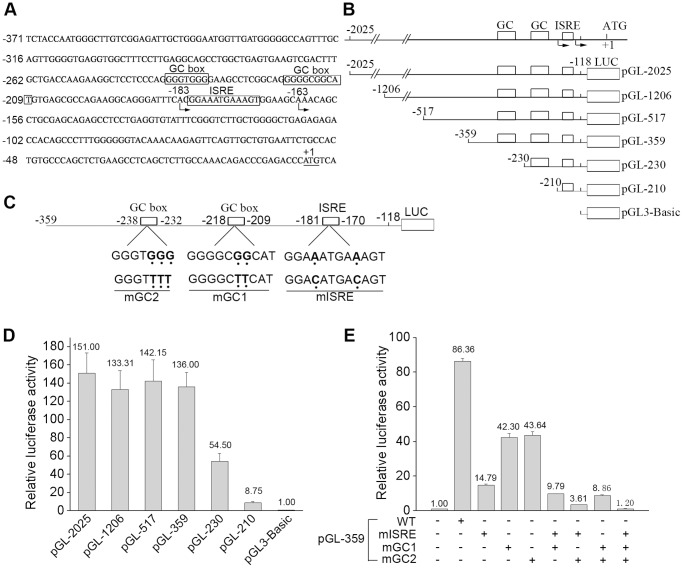
Identification and analysis of 5′ promoter region of IFP35 gene. (**A**) The ATG translational start site is underlined and the A is designed as +1. Arrowheads indicate the transcriptional start sites. The putative *cis*-elements are boxed. (**B**) Schematic representation of six 5′ upstream region deletions of IFP35 gene. (**C**) Schematic representation of the core promoter region of pGL-359. The mutations were constructed as indicated. (**D**) HeLa cells were transfected with the indicated promoter constructs. Luciferase assays were performed 48 h after transfection. β-gal activity was measured as a normalization control for the luciferase activity. (**E**) HeLa cells were transfected with the indicated wild type or mutant pGL-359 constructs. Luciferase assays were performed 48 h after transfection.

It was reported that the mRNA and protein levels of IFP35 could be induced by IFN-γ [1]. However, it has not yet been determined which elements and factors are involved in the induction of IFP35 expression by IFN-γ. In this study, we have identified a functional ISRE in the IFP35 promoter located at –181 bp from the start codon. Furthermore, we show that IRF-1 directly binds to this site and mediates IFN-γ-dependent transcriptional activation of IFP35 in HeLa cells.

## Results

### Isolation and Characterization of IFP35 Promoter

To obtain insights into the process of IFN-γ induction of the IFP35 gene, it is essential to characterize the IFP35 promoter. We first mapped the transcriptional start site of IFP35 by 5′-rapid amplification of cDNA ends (RACE) PCR. The PCR products were cloned into pMD18-T and sequenced. The sequences of the 10 clones obtained from the 5′-RACE products revealed two transcriptional start sites, located 163 (6 clones) and 183 (4 clones) nucleotides upstream of the translational start site, respectively (Fig. 1A). We then cloned the 5′ upstream region of the start sites (pGL-2025) and performed sequence analysis. The analysis using the TFSEARCH ver.1.3 revealed that the IFP35 promoter lacks canonical TATA and CAAT boxes. On the other hand, two conservative GC boxes (GC1 box and GC2 box) and a potential ISRE were found in the promoter region (Fig. 1A).

**Figure 2:**
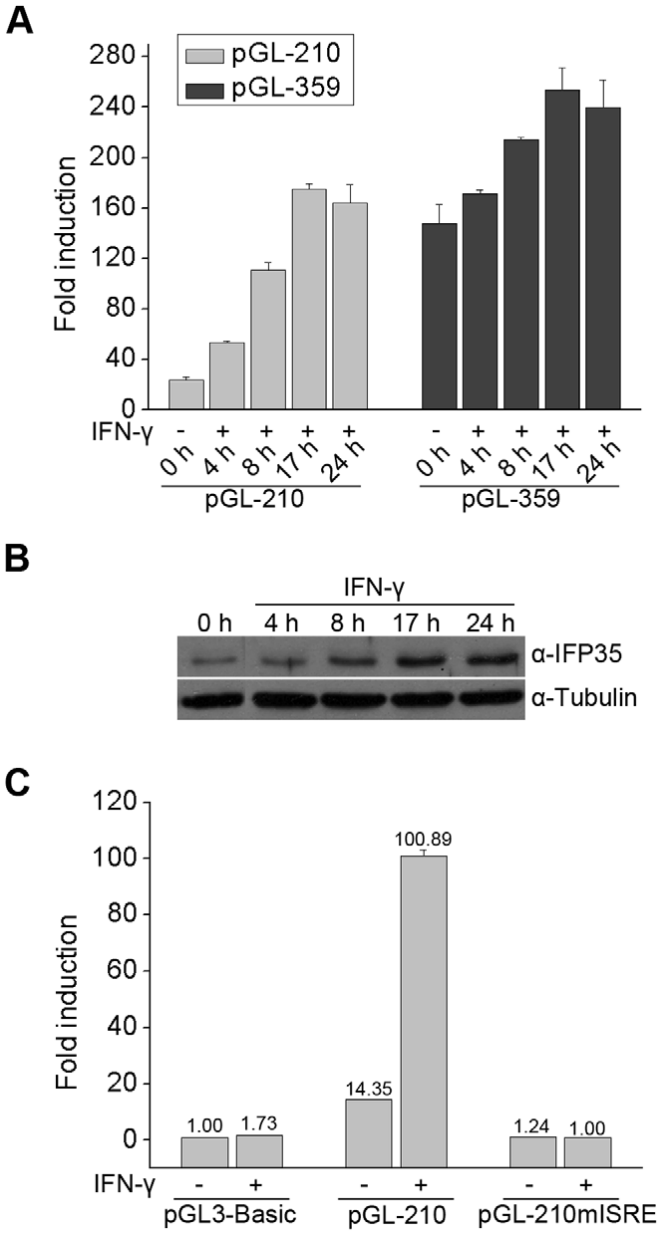
ISRE is responsible for IFN-γ induced IFP35 promoter activation. (**A**) HeLa cells were transfected with pGL-210 or pGL-359 and stimulated with IFN-γ (10 ng/ml) for different time periods. The response to IFN-γ is presented as fold induction relative to pGL3-Basic. (**B**) HeLa cells were stimulated with IFN-γ (10 ng/ml) for different time periods. The expression of IFP35 and α-tubulin was monitored by Western blot analysis. (**C**) HeLa cells were transfected with the pGL3-Basic, pGL-210 or pGL-210mISRE constructs. At 36 h after transfecion, cells were incubated with medium alone or with IFN-γ (10 ng/ml) for 12 h before luciferase assays were performed.

**Figure 3:**
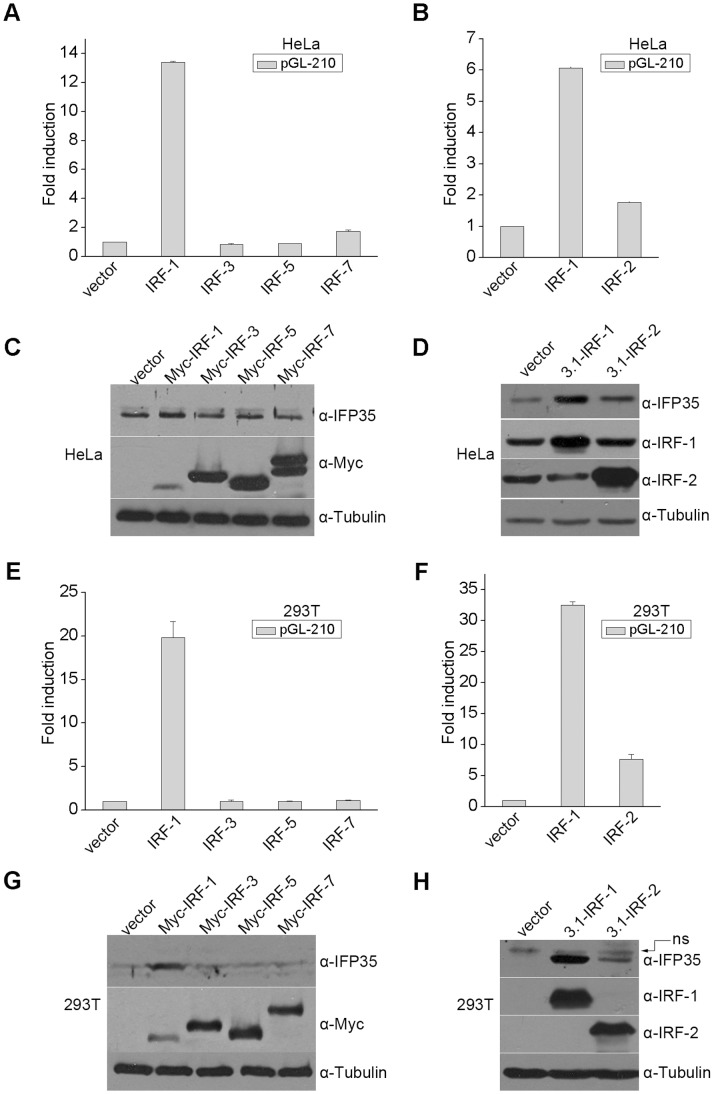
IRF-1 and IRF-2 upregulate IFP35 expression in HeLa and 293T cells. (**A and B**) HeLa cells were co-transfected with pGL-210 and the indicated expression plasmids. Luciferase assay was performed 48 h after transfection. (**C and D**) HeLa cells were transfected with the indicated expression plasmids. At 48 h after transfection, whole cell extracts were prepared and analyzed by Western blot. α-tubulin serves as an internal standard for normalization. (**E and F**) 293T cells were transfected with the indicated expression plasmids. Luciferase assay was performed 48 h after transfection. (**G and H**) 293T cells were transfected with the indicated expression plasmids. Western blot analysis was performed 48 h after transfection.

**Figure 4:**
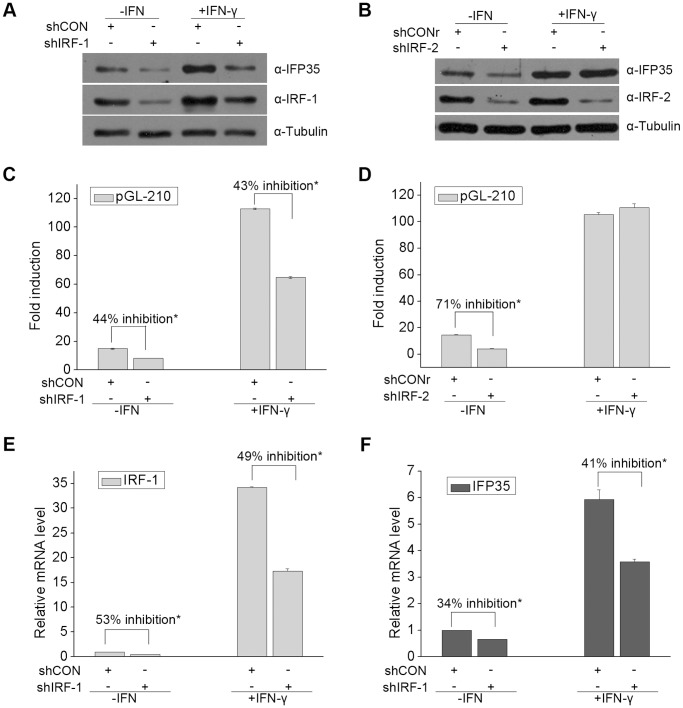
IRF-1 and IRF-2 differentially upregulate IFP35 expression in HeLa cell. (**A**) HeLa cells were transfected with shIRF-1 or shCON. At 36 h after transfection, cells were incubated for 12 h in the presence or absence of IFN-γ (10 ng/ml). Immunoblotting was then performed to examine the protein levels of IFP35, IRF-1 and α-tubulin. (**B**) The experiments were similarly performed as in (A) except that HeLa cells were transfected with shIRF-2 and shCONr. (**C and D**) HeLa cells were co-transfected with pGL-210 and the indicated shRNA expression plasmids. At 36 h after transfection, cells were cultured in the presence or absence of IFN-γ (10 ng/ml) for 12 h before luciferase assays were performed. The response to IFN-γ is presented as fold induction relative to unstimulated cells. Data are the mean and standard error from three experiments. ^*^P<0.05. (**E**) HeLa cells were transfected with shCON or shIRF-1. At 36 h after transfection, cells were incubated for 12 h in the presence or absence of IFN-γ (10 ng/ml) before real-time PCR was performed. Data are the mean and standard error from three experiments. ^*^P<0.05. (**F**) The experiments were similarly performed as in (E) except that IFP35 mRNA level was analyzed.

To determine the *cis*-acting regulatory elements involved in the basal activity of IFP35 promoter, promoter fragments with different length were generated and cloned into the pGL3-Basic vector (Fig. 1B). HeLa cells were transiently transfected with these reporter plasmids, and the relative luciferase activity was measured 48 h post transfection. As shown in Fig. 1D, basal transcription from all 4 constructs with deletions from −2025 to −359 appeared normal, but further deletion to position −230 (with the GC2 box deleted) resulted in a decrease of basal transcription activity of nearly 60%, and additional deletion to position −210 (with the GC1 box also deleted) resulted in a decrease of basal transcriptional activity of nearly 94%, only leaving a 8.75-fold increase in luciferase activity as compared to the control vector without a promoter sequence.

**Figure 5:**
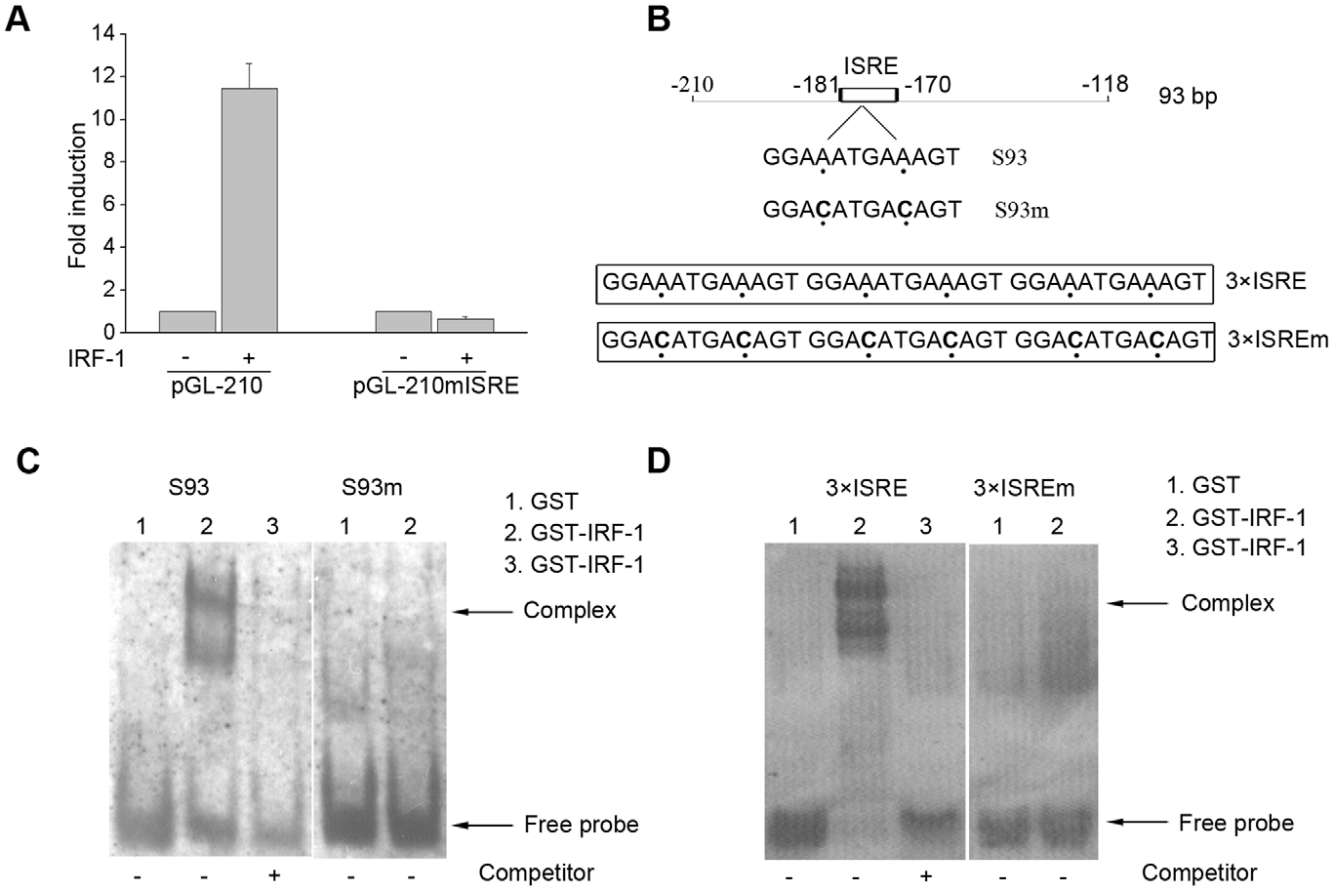
IRF-1 participates in IFP35 transcription by binding directly to the ISRE of the IFP35 promoter *in vitro*. (**A**) pGL-210 or pGL-210mISRE was co-transfected with the empty vector or the IRF-1 expression plasmid into HeLa cells. Luciferase assays were performed at 48 h after transfection. Data are the mean and standard error from three experiments. (**B**) Schematic diagram of the probes used in EMSA assay. (**C**) EMSA was performed with GST or GST–IRF-1. Digoxigenin-labeled S93 or S93m were used as probes. Arrowheads indicate DNA–protein complexes. (**D**) EMSA was performed with GST or GST–IRF-1. Digoxigenin-labeled 3×ISRE or 3×ISREm were used as probes. Arrowheads indicate DNA–protein complexes.

**Figure 6:**
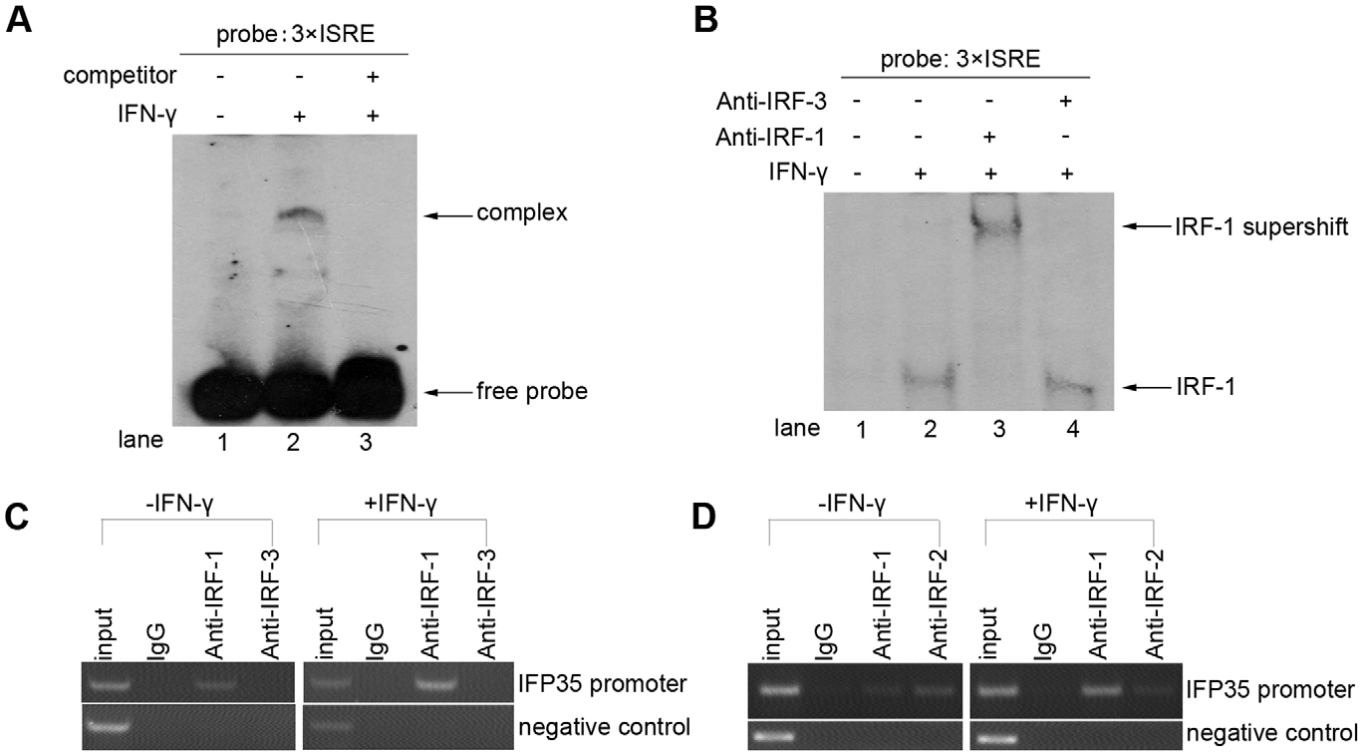
IRF-1 binds to IFP35 promoter upon IFN-γ treatment. (**A**) HeLa cells were either untreated or treated with IFN-γ (10 ng/ml) for 12 h. Nuclear extracts prepared from these cells were subjected to EMSA. The competitor represents 40×excess cold 3×ISRE. (**B**) Nuclear extracts (4 µg) from HeLa cells unstimulated or stimulated with IFN-γ (10 ng/ml) for 12 h were subjected to EMSA by using 3×ISRE probe. Supershift assays were performed by preincubating the nuclear extracts with 2 µg anti-IRF-1 or anti-IRF-3. The specific IRF-1 complex and supershifted complex were indicated by arrows. The free probes have run out of the gel. (**C**) HeLa cells were either untreated or treated with IFN-γ (10 ng/ml) for 12 h and processed for ChIP assays by using anti-IRF-1, anti-IRF-3 or control IgG. Precipitated DNA encompassing the IFP35 ISRE was then assayed by PCR. The negative control indicates a genomic fragment (+976 to +1337, relative to the translation start site) downstream of IL-7 promoter. (**D**) The experiment was similarly performed as in (C) except that anti-IRF-1 and anti-IRF-2 were used.

**Figure 7:**
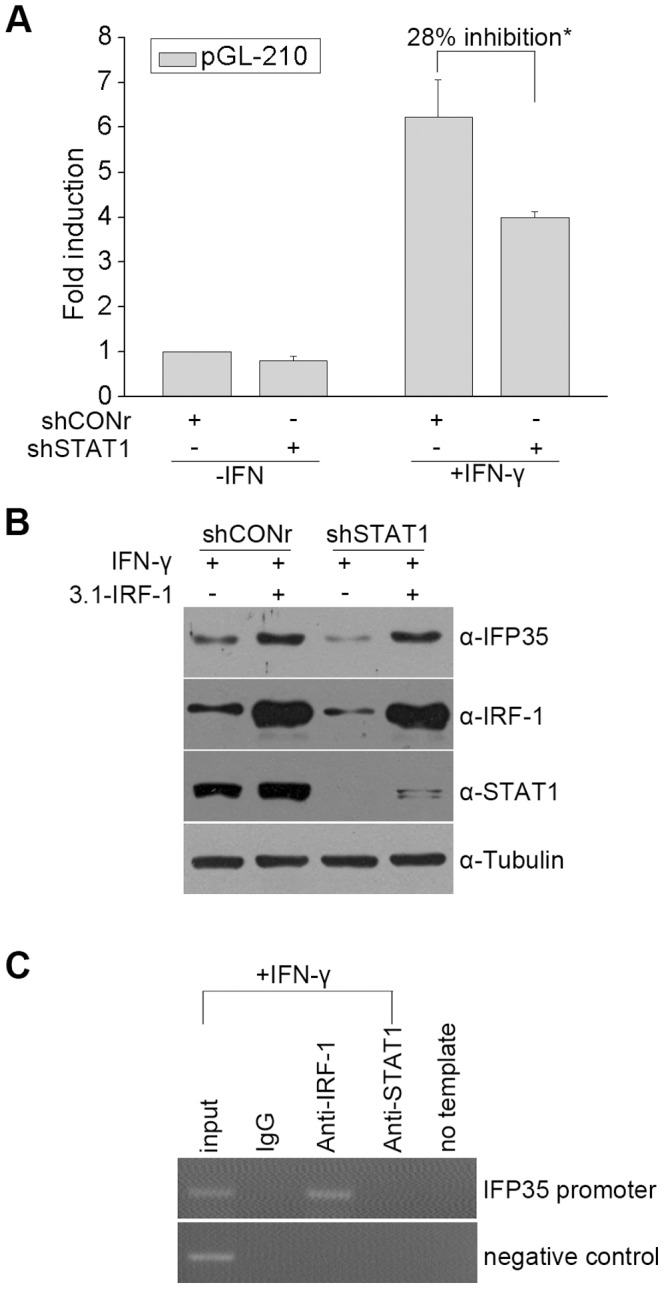
STAT1 is involved in IFN-γ-induced IFP35 expression in HeLa cells. (**A**) pGL-210 was co-transfected with shSTAT1 or shCONr into HeLa cells. At 36 h after transfection, cells were cultured in the presence or absence of IFN-γ (10 ng/ml) for 12 h before luciferase assays were performed. The response to IFN-γ is presented as fold induction relative to unstimulated cells. Data are the mean and standard error from three experiments. ^*^P<0.05. (**B**) HeLa cells stably expressing either shSTAT1 or shCONr were transfected with pCDNA3.1 or pCDNA3.1-IRF-1. At 36 h after transfection, cells were treated with IFN-γ (10 ng/ml) for 12 h before immunoblotting was performed. (**C**) HeLa cells were treated with IFN-γ (10 ng/ml) for 12 h and processed for ChIP assays by using control IgG, anti-IRF-1 or anti-STAT1. The negative control indicates a genomic fragment (+976 to +1337, relative to the translation start site) downstream of IL-7 promoter.

**Figure 8:**
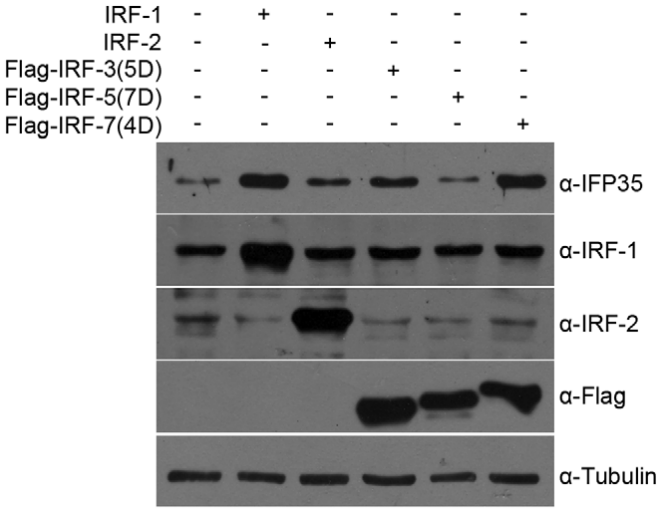
IFP35 expression is upregulated by constitutively active forms of IRF-3 and IRF-7 in HeLa cells. HeLa cells were transfected with the expression plasmids encoding IRF-1, IRF-2 and constitutively active forms of IRF-3, IRF-5 and IRF-7. At 48 h after transfection, whole cell extracts were prepared and Western blot analysis was performed with antibodies as indicated.

To further verify whether the two GC boxes and potential ISRE element are involved in the basal transcription of IFP35, we constructed several promoter mutants on the backbone of pGL-359 (Fig. 1C). As shown in Fig. 1E, mutations in the GC boxes decreased pGL-359 promoter activity by 51.02% (GC1 mutation) and 49.47% (GC2 mutation). Mutations in the ISRE decreased the basal activity of pGL-359 even to a lower level (by 82.87%). Furthermore, the mutation of two GC boxes and ISRE could synergistically reduce pGL-359 promoter activity (Fig. 1E, column 6, 7, 8). When all three elements were mutated, pGL-359 completely lost the promoter activity when compared to the empty vector, suggesting that all the three elements were essential for basal transcription of IFP35 in HeLa cells.

**Table 1:** Primers for PCR of IFP35 promoter fragments and site-directed mutagenesis.

Primer	Sequence (5′–3′)	Location
P210	AAAACGCGTATGTGAGCGCCAGAAGG	–210 to –194
P230	AAAACGCGTAAGCCTCGGCAGGGG	–230 to –216
P359	AAAACGCGTCTTGTCGGAGATTGCTG	–359 to –343
P517	AAAACGCGTGTGATCTGCCCGCCTG	–517 to –502
P1206	ATAGGTACCACAAGGCATGAGCCACTGCG	–1206 to –1187
P2025	TTTGGATCCATGCTTTGATTCCTAGCCCTG	–2025 to –1996
Plow	AAACTCGAGGCAAGACCCGAAATACACC	–136 to –118
mISREf	CAGGGATTTCACGGACATGACAGTGGAAGCAAACAG	
mISREr	CTGTTTGCTTCCACTGTCATGTCCGTGAAATCCCTG	
mGCf1	GGGAAGCCTCGGCAGGGGGCTTCATGTGAGCGCCAGAAGGCA	
mGCr1	TGCCTTCTGGCGCTCACATGAAGCCCCCTGCCGAGGCTTCCC	
mGCf2	GCTCCTCCCAGGGGTTTTGAAGCCTCGGCAGGG	
mGCr2	CCCTGCCGAGGCTTCAAAACCCCTGGGAGGAGC	

### ISRE Consensus Binding Site is Critical for IFN-γ Induced IFP35 Promoter Activity

In order to determine the IFN-γ responsiveness of IFP35 promoter, HeLa cells were transfected with pGL-210 or pGL-359, and then treated with IFN-γ for different time periods. As shown in Fig. 2A, the luciferase activity of pGL-210 and pGL-359 increased after 4 h of IFN-γ stimulation and reached the maximum level after 17 h of treatment.

To compare the time course of the induction of the reporter construct and the induction of IFP35 protein upon IFN-γ treatment, we performed Western blot assay for IFP35 protein in HeLa cells treated with IFN-γ for different time periods. The amount of IFP35 protein also increased after 4 h of stimulation and reached the maximum level at 17 h in parallel to that of the promoter induction (Fig. 2B), suggesting that both pGL-210 and pGL-359 can account for the induction of IFP35 protein. Additionally, it revealed that pGL-210 had significantly higher luciferase fold induction as compared with pGL-359, we speculated that some factors binding at −210 to −359 may partially inhibit the function of ISRE. Therefore, we conducted the following experiments using the pGL-210 promoter construct to focus on factors that activate ISRE.

The results above indicate that a potential ISRE site is present in IFP35 promoter (Fig. 1D). To identify whether this ISRE was responsible for IFN-γ induction of pGL-210 activity, we mutated the ISRE on pGL-210. The mutated construct, named pGL-210mISRE, completely lacked IFN-γ responsiveness upon transfection into HeLa cells, suggesting that the ISRE site is functional and critical for the induction of IFP35 promoter by IFN-γ (Fig. 2C).

### IRF-1 and IRF-2 Activate IFP35 Promoter and Increase IFP35 Expression

Members of the IRF family can bind to ISRE and exert transcriptional regulation of the target genes [14]. To determine which IRF family member activates the IFP35 promoter via ISRE, we co-transfected pGL-210 with plasmids expressing IRF-1, IRF-2, IRF-3, IRF-5 or IRF-7 to HeLa and 293T cells, and examined pGL-210 promoter activity. It was found that over-expression of IRF-1 led to a dramatic increase in pGL-210 promoter activity in both HeLa and 293T cells (Fig. 3A, 3B, 3E and 3F). Although IRF-2 and IRF-7 both slightly activated pGL-210 in HeLa cells, only IRF-2 significantly activated pGL-210 in 293T cells (7.7-fold) (Fig. 3A, 3B, 3E and 3F).

To further determine which IRF activates the endogenous IFP35 expression, Western blot analyses were performed. As expected, the IFP35 protein level was dramatically increased by IRF-1 in 293T and HeLa cells. IRF-2 also led to a moderate upregulation of IFP35 expression in both cell lines, whereas other IRFs exerted no effect (Fig. 3C, 3D, 3G and 3H).

### IRF-1 and IRF-2 Differentially Upregulate IFP35 Expression Upon IFN-γ Treatment

Our results above suggested that IRF-1 and IRF-2 are potential activators of IFP35. To determine whether IRF-1 and IRF-2 are involved in IFN-γ inducible IFP35 expression, we depleted IRF-1 and IRF-2 by shRNA in HeLa cells. As shown in Fig. 4A, when HeLa cells were transiently transfected with shIRF-1, both basal and IFN-γ-induced expression of IRF-1 were effectively suppressed. Consistent with this result, in cells transfected with shIRF-1, basal and IFN-γ-induced IFP35 protein expression was obviously inhibited. In contrast, silencing IRF-2 expression resulted in the suppression of basal IFP35 expression, but not the IFN-γ-inducible expression (Fig. 4B). In luciferase assay, we observed that basal promoter activity of pGL-210 was inhibited in cells depleted of IRF-1 (by 44%) or IRF-2 (by 71%). In addition, in cells transfected with shIRF-1, IFN-γ-dependent pGL-210 induction is reduced by 43%, whereas no inhibition was seen in cells depleted of IRF-2 (Fig. 4C and 4D). We further demonstrated that shIRF-1 decreased IFP35 mRNA level in both unstimulated (by 34%) and IFN-γ-stimulated cells (by 41%) (Fig. 4F). The IRF-1 knockdown efficiency is shown in Fig. 4E. Collectively, these data indicate that IRF-1 is involved in both basal and IFN-γ-induced IFP35 expression, while IRF-2 predominantly maintains the constitutive expression of IFP35.

### IRF-1 is Recruited to the ISRE Site of IFP35 Promoter Upon IFN-γ Treatment

To further verify whether IRF-1 can activate IFP35 promoter via binding to ISRE, we co-transfected IRF-1 with pGL-210 or pGL-210mISRE into HeLa cells. The ISRE mutant (pGL-210mISRE) completely abolished the induction by IRF-1 (Fig. 5A), indicating the important role of this element in IRF-1-mediated IFP35 transcription. To further analyze the involvement of this ISRE motif, we used the probes containing this element (S93 and 3×ISRE) (Fig. 5B) and purified GST or GST-IRF-1 proteins for EMSA. A single shifted band was detected when the probes was incubated with purified GST-IRF-1 protein, whereas no band was detected when purified GST protein was added. The band was abolished by addition of 100-fold concentrations of unlabelled probes as the competitors (Fig. 5C and 5D). In contrast, when S93 and 3×ISRE were mutated, no complex was formed (Fig. 5C and 5D). These findings suggest that IRF-1 directly binds to the IFP35 promoter and regulates its activity.

We next investigated whether endogenous IRF-1 bound to the ISRE site of IFP35 promoter after IFN-γ treatment *in vitro*. For this purpose, we performed gel supershift analysis. A single shifted band was detected when 3×ISRE was incubated with nuclear extracts derived from IFN-γ-stimulated HeLa cells (Fig. 6A, lane 2), and this band was competed by 40× excess unlabeled ISRE (Fig. 6A, lane 3). Supershift analysis revealed that the shifted band was recognized by anti-IRF-1 antibody (Fig. 6B, lane 3), but not by anti-IRF-3 antibody (Fig. 6B, lane 4).

To further verify the interaction between endogenous IRF-1 and IFP35 promoter, ChIP assay was performed. PCR primers were used to amplify DNA fragment corresponding to the region −370 to −45 (relative to the translation start site), which contains the ISRE site encompassing from −181 to −170 (relative to the translation start site). As shown in Fig. 6C & 6D, IRF-1 and IRF-2 bound to the IFP35 promoter in the absence of IFN-γ stimulation, whereas control IgG and anti-IRF-3 did not immunoprecipitate any factor bound to the IFP35 promoter, indicating that the occupancy of IRF-1 and IRF-2 on the promoter was specific. Additionally, we observed that IRF-1 but not IRF-2 was further recruited to IFP35 promoter after IFN-γ treatment (Fig. 6D), suggesting that IFP35 promoter is constitutively occupied by IRF-1 and IRF-2, and the interaction with IRF-1 is further enhanced upon IFN-γ treatment. Besides, it was reported that IRF-1 and IRF-2 could not bind to the region far downstream of IL-7 promoter (+976 to +1337, relative to the translation start site) [24]. Therefore, this fragment was amplified as a negative control in our ChIP assay to certify that binding of IRF-1 and IRF-2 to IFP35 promoter was specific (Fig. 6D).

### STAT1 is Involved in IFP35 Induction by IFN-γ

The expression of IRF-1 is regulated by STAT1 homodimer upon IFN-γ treatment [18]. Thus, we speculate that knock down of STAT1 may decrease IRF-1 induction by IFN-γ, which may further inhibit IFN-γ-dependent IFP35 expression. As shown in Fig. 7A, shSTAT1 decreased IFN-γ-induced pGL-210 activity by 28%. Furthermore, HeLa cells stably expressing STAT1-target or control shRNA were treated with IFN-γ for 12 h. Western blot revealed that IRF-1 and IFP35 induction by IFN-γ was inhibited in cells stably expressing shSTAT1, and this inhibition was rescued by IRF-1 over-expression (Fig. 7B). These results demonstrated that STAT1 plays a role in IFN-γ-mediated IFP35 induction. To exclude the possibility that STAT1 may directly regulate IFP35 expression by binding to IFP35 promoter, we performed ChIP assay. As shown in Fig. 7C, STAT1 did not bind to our defined IFP35 promoter in IFN-γ-treated HeLa cells. These results clearly confirmed our speculation that IFN-γ induces IRF-1 expression via STAT1, and the enhanced IRF-1 expression further lead to an increase of IFP35 expression.

### Constitutively Active IRF-3 and IRF-7 Upregulate IFP35 Expression in HeLa Cells

IRF-3, IRF-5 and IRF-7 are direct transducers of virus-mediated signalling and play an essential role in the expression of cytokines and chemokines. In unstimulated cells, they are predominantly present in the cytoplasm in inactive forms. Following pathogenic infection, they are phosphorylated and translocate into the nucleus where they regulate transcription by binding to the promoter regions of target genes [15]. From the results presented above, we found that over-expression of IRF-7 can slightly activate IFP35 promoter. However, IRF-7 is predominantly induced by type I IFN, but not IFN-γ [25]. In addition, it needs to be post-translationally modified by stimulators, such as virus infection. Thus, we speculate that IRF-7 may not be a key regulator in IFN-γ-inducible IFP35 expression, although we cannot rule out the possibility that active IRF-7 may still contribute to IFP35 expression. To test this possibility, we transfected constitutively active IRF-3, IRF-5 and IRF-7 into HeLa cells [26], [27]. Interestingly, both constitutively active IRF-3 and IRF-7 dramatically upregulated IFP35 protein level, whereas constitutively active IRF-5 exerted little effect on IFP35 expression (Fig. 8). These results indicated that IRF-3 and IRF-7 may also be potential IFP35 activators.

## Discussion

IFP35 was first isolated as an IFN-induced protein [1]. As an IFN-stimulated gene, IFP35 can confer resistance to BFV infection in HeLa cells [7]. Besides, it is observed that expression of IFP35 is significantly down-regulated in the T cells of Sezary Syndrome patients, indicating that IFP35 may be implicated in tumor suppression [3]. The molecular function of IFP35 mandates a detailed study of its expression regulation. In the present study, we set out to examine the mechanism of IFN-γ-induced upregulation of IFP35 expression.

We initially characterized the transcription start sites of IFP35 and cloned the IFP35 promoter. Similar to many other IFN-induced genes, such as Sp100 [28], GBP [9], and IFITM1 [29], our results revealed that transcription from the IFP35 promoter is also initiated at heterogeneous transcription start sites. Analysis of the IFP35 promoter revealed two GC boxes and an ISRE site, but no consensus sequence for a canonical TATA box. This is not completely surprising, as utilization of multiple transcription start sites is frequently observed in the regulation of genes whose promoters are driven by GC boxes instead of TATA boxes [30]. In addition, the use of a TATA-less promoter may contribute to cell type-specific expression of IFP35, and therefore define the specific cell types that respond to a systemic IFN signal. For example, cells expressing different levels or different relative contribution of the Sp family of transcription factors, which are known to bind to GC boxes, may exhibit different basal and induced activities of IFP35 promoter. This hypothesis will need to be tested in future investigations. ISREs control the expression of most IFN-α regulated genes and some IFN-γ regulated genes. ISRE is also known to be involved in constitutive expression of some ISGs including TAP2 and ISG20 [19], [31]. Our results revealed that mutagenesis of the ISRE in IFP35 promoter greatly decreased its basal activity and completely abolished its response to IFN-γ, indicating that this ISRE is also involved in both constitutive and IFN-induced transcription.

Recent evidence indicates that IRF-1 and IRF-2 may bind to the same promoter region and cooperatively activate gene transcription [24], [32]. In our study, we found that both IRF-1 and IRF-2 bound to the IFP35 promoter, suggesting that they may cooperate in the regulation of IFP35 expression. However, the basal expression of IRF-2 was not affected by IFN-γ treatment, suggesting that IRF-2 alone was not sufficient to activate IFP35 expression. In contrast, IRF-1 expression was significantly increased upon IFN-γ treatment. Thus, these results further indicated that IRF-1 not only controls the constitutive IFP35 expression, but also plays a predominant role in IFN-γ dependent IFP35 expression.

IRF-1 is reported to be upregulated by IFN-γ and serves as a secondary transcriptional activator of many IFN-γ regulated genes via the ISRE site. It plays various roles in multiple biological processes by regulating downstream genes involved in antiviral defense (e.g., ISG20 [19] and PKR [33]), apoptosis (e.g., Caspase 8 [34]), and inflammation (e.g., IL-7 [24] and RANTES/CC15 [20]). In this study, our results revealed that IFN-γ and IRF-1 were able to stimulate IFP35 expression via the ISRE site. This led us to propose that IFN-γ activates IFP35 by enhanced recruitment of IRF-1 to the IFP35 ISRE site, which was confirmed by using multiple approaches. First, IRF-1 is recruited to the ISRE site of IFP35 promoter *in vitro* after IFN-γ treatment in HeLa cells, as demonstrated by gel supershift experiments. Second, IRF-1 could bind to the IFP35 promoter, and treatment with IFN-γ increased the amount of IRF-1 recruited to IFP35 promoter, as demonstrated by ChIP experiment. Third, blocking IRF-1 expression by using a siRNA strategy inhibits the induction of IFP35 synthesis by IFN-γ.

Phosphorylated STAT1 homodimer is reported to activate genes by directly binding to GAS of the promoters upon IFN-γ treatment [35], [36]. However, we did not find GAS in IFP35 promoter, and our ChIP assay demonstrated that STAT1 did not associate with our defined IFP35 promoter upon IFN-γ treatment. Thus, a direct influence of STAT1 on the IFP35 expression is unlikely. On the contrary, our results revealed that knock down of STAT1 decreased IRF-1 expression, along with decreased IFP35 expression. This supports our hypothesis that STAT1 is essential for IRF-1 activation and the enhanced IRF-1 expression is sufficient to increase IFP35 expression.

In our study, we also tested the effect of IRF-3, IRF-5 and IRF-7 on IFP35 expression. IRF-3, IRF-5 and IRF-7 are known to play essential roles in virus-induced type I IFN gene expression. Although IRF-3 is constitutively expressed in all cell types, IRF-5 and IRF-7 are predominantly expressed in cells of lymphoid origin and can be further induced by type I IFN [25], [37], [38], [39], [40]. Besides, they all need to be phophorylated to become active. Thus, constitutively active forms of the IRFs were used in our experiment to mimic activated forms of the proteins in virus-infected cells [26], [27]. Our results revealed that constitutively active IRF-3 and IRF-7 significantly increased IFP35 protein level. Although further investigations are needed to determine whether the IRFs are acting directly by binding to IFP35 promoter or indirectly through induction of type I IFN, the results did give us some hints that IFP35 may be modulated in multiple ways under certain circumstances, such as virus infection.

In summary, this study demonstrates that IFP35 expression induced by IFN-γ can be regulated at the transcriptional level. This transcriptional regulation by IFN-γ is the result of the recruitment of the transcription factor IRF-1 to the ISRE site in the IFP35 promoter. However, whether other factors are also involved in IFN-γ induction of IFP35 expression still needs further investigations.

## Materials and Methods

### Materials

IFN-γ was purchased from Millipore. Antibodies against IRF-1, IRF-2, IRF-3, and horseradish peroxidase-conjugated secondary antibodies (Santa Cruz Biotechnology); STAT1 (Cell signaling); Myc (Millipore) and α-tubulin (Sigma) were purchased from the indicated sources. Antibody against IFP35 was generated by immunization of mice with His-IFP35 fusion protein. GST and the GST-IRF-1 fusion protein were purified with glutathione-Sepharose beads (GE Healthcare), and His-IFP35 fusion protein was purified with the nickel-nitrilotriacetic acid agarose chromatography (GE Healthcare). Protein concentrations were determined by Bradford method.

### Plasmids Construction

The 5′-flanking region of the IFP35 gene (1,908 bp in length) was amplified by PCR from genomic DNA isolated from HeLa cells. The progressive 5′-deletion constructs pGL-2025, pGL-1206, pGL-517, pGL-359 (GenBank accession number: JX878502), pGL-230 and pGL-210 of IFP35 promoter were created by PCR using primers listed in table 1. pGL-359 mutant plasmids were generated by site-directed mutagenesis kit (Toyobo) and primers used were listed in table 1 (mISREf and mISREr for ISRE mutation; mGCf1 and mGCr1 for GC box1 mutation; mGCf2 and mGCr2 for GC box2 mutation). The mammalian expression plasmids pCMV-HA-IRF-1, pCDNA3.1-IRF-3, pCDNA3.1-IRF-5 and pCDNA3.1-IRF-7 were obtained from Pamela I. Österlund (National Public Health Institute, Helsinki, Finland) [41]. pCDNA3.1-IRF-1, pCDNA3.1-IRF-2, pCMV-myc-IRF-1, pCMV-myc-IRF-3, pCMV-myc-IRF-5 and pCMV-myc-IRF-7 plasmids were constructed by insertion of cDNA into the pCDNA3.1 (+) or pCMV-Tag3B vectors. GST-IRF-1 was generated by inserting IRF-1 cDNA into the pGEX-6p-1 vector. pFLAG-IRF-3(5D), pFLAG-IRF-5(7D) and pFLAG-IRF-7(4D) were kindly provided by Rongtuan Lin (McGill University, Montreal, Canada). shIRF-1 and shCON were constructed using pBSU6 vector in accordance with the manufacturer’s protocols. The control shRNA, shCON was designed based on the sequence: 5′-AATTCTCCGAACGTGTCACGT-3′, without specific target in cells [42]. The IRF-1 target sequence was: 5′-GGGGTACCTACTCAATGAACCT-3′. shCONr, shIRF-2 and shSTAT1 were constructed using the pSIREN-RetroQ vector (Clontech). Target sequence for shCONr was the same as shCON. Target sequence for STAT1 was: 5′-GGAAAAGCAAGCGTAATC. T-3′; IRF-2∶5′-GCAAGAACCAGTTGAGTCA-3′. The sequences of all of the constructs were confirmed by sequencing.

### Cell Culture and Transfection

HeLa and 293T cells (maintained in our lab) were grown in Dulbecco’s modified Eagle’s medium (Gibco, Gaithersburg, MD, USA) supplemented with 10% fetal calf serum at 37°C in humidified air containing 5% CO_2_. Transfection was carried out with polyethyleneimine (PEI) reagent (Polysciences) [43].

### Generation of Stable shRNA-expressing HeLa Cells

For generation of VSV-G pseudo-typed retrovirus, 293T cells were transfected with shSTAT1 or shCONr along with plasmids encoding gag-pol and VSV-G proteins. Supernatants were harvested after 48 h. HeLa cells were infected with the retroviruses by spinning for 30 min at 1,500 rpm, in presence of 5 µg/ml polybrene. The virus was removed 12 h after the infection and fresh medium was added. At 48 h after infection, cells were selected with 2 µg/ml puromycin.

### 5′-Rapid Amplification of cDNA Ends (RACE)

5′-RACE was used to characterize the 5′-end of IFP35 transcript (Ambion Firstchoice RLM-RACE Kit in accordance with the manufacturer’s instructions). Total RNA was extracted from HeLa cells with Qiagen RNeasy Mini Kit. Nested PCR was carried out with two pairs of primers. The outer and inner RACE sense primers were provided by the kit, the sequence of the gene-specific primer used for the first round PCR was: 5′-GCCACTTTGGGGTCATCAAAGGT-3′, and primer used for the second round of PCR was: 5′-GGCACTGAAAATGGGACCTTGTCTTTG-3′. PCR products were cloned into the pMD18-T vector for analysis and DNA sequencing.

### Electrophoretic Mobility Shift Assays

EMSA was carried out using DIG Gel Shift Kit (Roche). Probes for S93 (−210 to −118, relative to the translation start site) and S93m were generated by PCR from pGL-210 and pGL-210mISRE. 3×ISRE (5′-CGCGTCACGGAAATGAAAGTGGAAATGAAAGTGGAA ATGAAAGTGGAC-3′) and 3×ISREm (5′-CGCGTCACGGACATGACAGTGGACATGAC AGTGGACATGACAGTGGAC-3′) were synthesized and annealed with the complementary strand. The purified GST, GST-IRF-1 or Nuclear extracts were incubated with digoxigenin (DIG)-labelled probe in binding buffer for 20 min at room temperature, the DNA–protein complexes were separated on 6% non-denaturing polyacrylamide gel and subjected to visualization by anti-DIG-AP antibody according to the protocol.

Supershift assays were performed by incubating nuclear extracts with 2 µg of anti-IRF-1 or anti-IRF-3 for 30 min on ice before addition of the labelled probe. The reaction mixtures were then electrophoresed and processed as describe above.

### Quantitative Real-time RT-PCR

Total RNA was extracted from cells using the Qiagen RNeasy Mini Kit. Real-time RT-PCR was conducted with the SYBR green real-time PCR mastermix (Toyobo) on the IQ5 Multicolor real-time PCR Detection system (Bio-Rad). The PCR primers used were, GAPDH: sense, 5′-AACAGCGACACCCACTCCTC-3′ and anti-sense, 5′-CATACCAGGAAATGAGC. TTGACAA-3′; IFP35: sense, 5′-AACGGAGGTGGCGATGTGGAC-3′ and anti-sense, 5′-CACTGTGAACTGGCCGATTTGG-3′; IRF-1: sense, 5′-GCCCTCCACCTCTGAAGCTA. CAAC-3′ and anti-sense, 5′-TCTGTGAAGACACGCTGTAGACTC-3′. The expression level of IFP35 and IRF-1 was normalized to GAPDH by using the 2^−ΔΔCT^ method [44].

### Chromatin Immunoprecipitation (ChIP) Assay

HeLa cells were treated or untreated with IFN-γ for 12 h. The ChIP procedure was performed in accordance with the manufacturer’s instructions (Millipore), except that the eluted DNA was precipitated by phenol-chloroform extraction and ethanol precipitation in the presence of glycogen, which significantly increases the efficiency of extracted DNA [45], [46]. PCR primers used for amplifying the IFP35 promoter were: sense, 5′-CTACCAATGGGCTT. GTCGGAGATT-3′ and anti-sense: 5′-CACAGTGGCAGAATTCACAGCAAC-3′. Primers used for the negative control were: sense, 5′-GCTCTTTCTTTTGATGGCTACTCCG-3′ and anti-sense, 5′-TAGCCCATGATTCATATAACTGTGC-3′ [24].

### Immunoblotting

Cells were lysed and proteins were separated by 12% SDS-PAGE (polyacrylamide gel electrophoresis). The gels were subsequently blotted onto polyvinylidene difluoride membrane (Millipore). The membranes were blocked and incubated with primary antibodies and then with horseradish peroxidase-conjugated secondary antibodies. Specific proteins were visualized with enhanced chemiluminescence detection reagent (Millipore).

### Luciferase Reporter Assay

Cells were transfected with the luciferase plasmids and a β-galactosidase expressing plasmid. The luciferase activity was measured with a luciferase reporter assay system (Promega) and normalized to β-galactosidase activity.
